# Editorial: Physio-logging in marine animals: recent advances and future directions

**DOI:** 10.3389/fphys.2025.1675117

**Published:** 2025-08-28

**Authors:** Kagari Aoki, J. Chris McKnight, Takashi Kitagawa

**Affiliations:** ^1^ Faculty of Life and Environmental Sciences, Teikyo University of Science, Uenohara, Japan; ^2^ Sea Mammal Research Unit, School of Biology, Faculty of Science, University of St Andrews, St. Andrews, United Kingdom; ^3^ Graduate School of Frontier Sciences, The University of Tokyo, Kashiwa, Japan

**Keywords:** physio-logging, bio-logging, electrocardiograms (ECGs), thermal adaptation, oxidative stress, neurologgers

Integrating physiological data, such as heart rate, respiration, body temperature, and stress hormone levels with specific behavioral data, allows us to unravel the underlying mechanisms that may directly or indirectly limit, drive, and/or affect certain actions. These insights contribute to our understanding of the process of adaptive evolution and provide vital information for interpreting how behavior and population dynamics interact with extrinsic *and* intrinsic factors. Building upon the foundations of traditional bio-logging and its capacity to remotely measure and understand behavior and distribution, as well as environmental characteristics, physio-logging enables researchers to investigate physiological processes. This linkage between behavior, distribution, environment, and intrinsic physiology is crucial for the fields of ecology and conservation physiology (Cooke et al., 2021; Fahlman et al., 2021).

Physio-logging has provided valuable insight into how animals respond to various environmental factors, such as the behavioral thermal adaptation of Pacific bluefin tuna *Thunnus thynnus orientalis* (e.g., Kitagawa et al., 2000; Kitagawa et al., 2022) and the behavioral response of homing chum salmon *Oncorhynchus keta* to varying ambient temperature structures (Kitagawa et al., 2016). Physio-logging can also provide the necessary tools for conservation management, which will contribute toward reducing the impacts of anthropogenic disturbances on species. For example, assessment of stress levels, such as measuring corticosterone, cortisol or heart rate, may help evaluate the impact of anthropogenic disturbance (e.g., Thompson et al., 2014; Lyamin et al., 2016). This potential has been demonstrated in recent studies across taxa: for example, tissue perfusion and behavioral conflict have been documented in grey seals *Halichoerus grypus* using physio-logging tools (McKnight et al., 2019). Similarly, Ponganis 2021 applied heart rate loggers to air-breathing animals such as a blue whale *Balaenoptera musculus* and emperor penguins *Aptenodytes forsteri* to monitor physiological effort during deep dives, revealing thresholds in oxygen management relevant to survival limits under environmental stress. Near-infrared spectroscopy has also been proposed by Ruesch et al. (2022) as a non-invasive tool to assess blood oxygenation in marine mammals, thereby opening new avenues for evaluating stress and health status under conditions of human care or environmental disturbance (Fahlman et al., 2021; Ruesch et al., 2022). This emerging era of physio-logging will enable long-term studies aimed at improving our understanding of fundamental physiological function, health, welfare or wellbeing of animals (e.g., Abe et al.; Naveed Yousaf et al., 2022) and humans, as well as their responses to environmental and/or anthropogenic changes. The current Research Topic, “Physio-logging in Marine Animals: Recent Advances and Future Directions”, introduces diverse application examples in the field of physio-logging.

In this Research Topic, physio-logging techniques are applied to diverse taxa, including sea turtles, seabirds and teleost fish. For instance, Saito et al. introduced non-invasive methods to measure electrocardiograms (ECGs), which provide heart rate data in green sea turtles—an important step toward understanding their physiological adaptations to the environment. Narazaki et al. and Makiguchi et al. extended these approaches to reproductive context in loggerhead turtles *Caretta caretta* and chum salmon, respectively, and provided insights into autonomic nervous system activity through variations in heart rate during spawning events. Abe et al. and Aoki et al. explored how thermal physiology shapes the behavior and thermoregulatory capacity of tunas. They emphasized the importance of both heat retention and production in thermal adaptation, offering insight into how tunas cope with environmental variability through physiological and behavioral mechanisms. Koyama et al. and Mizutani et al. investigated how foraging behavior and oxidative stress reflect the physiological condition and adaptive strategies in seabirds. Their work highlights the importance of integrating behavioral, oxidative stress, and environmental data to understand energy allocation, reproductive trade-offs, and species responses to ecological pressures. The other paper summarized findings from studies applying neurologgers to teleost fish, revealing various spatial-cognition cells in regions of the telencephalon analogous to the mammalian hippocampus that are deeply involved in spatial navigation (Takahashi et al.). These contributions demonstrate the transformative capacity of physio-logging to uncover physiological correlates of behavior across a variety of taxa and life-history stages.

In the future, as new sensors such as brain wave sensors are developed and big data recording and analysis technologies evolve, physio-logging will continue to evolve as a tool for measuring the physiological functions of marine organisms and will contribute to our understanding of the ecology of organisms on Earth, including ourselves.
